# Supplementary material for: Simultaneous Quantification of Organic Acids in Tamarillo (Solanum betaceum) and Untargeted Chemotyping Using Methyl Chloroformate Derivatisation and GC-MS
Source: Molecules. 2022 Feb 15;27(4):1314. doi: 10.3390/molecules27041314 (PMC8875926; doi:10.3390/molecules27041314)
Supplement: Supplementary file 1 [file molecules-27-01314-s001.zip › molecules-1498998-supplementary.pdf]

Supplementary Table S1: Summary data for the absolute quantitative analysis of sixteen organic acids in tamarillo peel and pulp by derivatisation with methyl chloroformate and GC-MS.

compound	CAS	mz	RT	linear range (mg L^{-1})	r^2	recovery
malonic acid	141-82-2	101	10.365	0 - 800	0.991	0.83
succinic acid	110-15-6	116	12.114	0 - 800	0.997	0.98
lactic acid	10326-41-7	131	12.265	0 - 50	0.992	0.53
fumaric acid	110-17-8	144	12.286	0 - 800	0.999	0.88
benzoic acid	65-85-0	106	12.645	0 - 200	0.991	0.77
itaconic acid	97-65-4	127	13.201	0 - 800	0.997	2.62
2-hydroxybutyric acid	5094-24-6	117	13.449	0 - 800	0.997	0.72
malic acid	97-67-6	103	14.326	0 - 800	0.999	0.87
para-toluic acid	99-94-5	119	14.506	0 - 800	0.999	1.02
adipic acid	124-04-9	144	15.325	0 - 200	0.992	0.77
trans-cinnamic acid	140-10-3	163	17.124	0 - 800	0.996	0.83
cis-aconitic acid	585-84-2	185	18.142	0 - 800	0.991	1.11
citric acid	77-92-9	153	18.931	0 - 800	0.999	1.57
3-hydroxybenzoic acid	99-06-9	210	19.684	0 - 800	0.992	0.85
vanillic acid	121-34-6	209	22.024	0 - 800	0.991	0.89
syringic acid	530-57-4	239	24.132	0 - 800	0.992	0.87

Supplementary Table S2: Summary of the statistical analysis of the untargeted data.

PC	pairwise	p-value	transform	Amber peel vs Amber pulp	Amber peel vs Laird Large peel	Amber peel vs Laird Large pulp	Amber peel vs Mulligan peel	Amber peel vs Mulligan pulp	Amber pulp vs Laird Large peel	Amber pulp vs Laird Large pulp	Amber pulp vs Mulligan peel	Amber pulp vs Mulligan pulp	Laird Large peel vs Laird Large pulp	Laird Large peel vs Mulligan peel	Laird Large peel vs Mulligan pulp	Laird Large pulp vs Mulligan peel	Laird Large pulp vs Mulligan pulp
1	sinapic acid	1.03E-12	none	TRUE	TRUE	TRUE	FALSE	TRUE	TRUE	FALSE	TRUE	FALSE	TRUE	TRUE	TRUE	TRUE	FALSE
1	cis-aconitic acid	1.32E-11	inverse	TRUE	TRUE	TRUE	TRUE	TRUE	FALSE	FALSE	TRUE	FALSE	FALSE	TRUE	FALSE	TRUE	FALSE
1	glycine	5.54E-10	none	TRUE	FALSE	TRUE	TRUE	TRUE	TRUE	FALSE	TRUE	FALSE	TRUE	TRUE	TRUE	TRUE	TRUE
1	4-aminobutyric acid	2.75E-09	inverse	TRUE	TRUE	TRUE	TRUE	TRUE	TRUE	FALSE	TRUE	FALSE	FALSE	FALSE	FALSE	TRUE	FALSE
1	lysine	1.33E-08	none	TRUE	FALSE	TRUE	FALSE	TRUE	TRUE	TRUE	TRUE	FALSE	TRUE	FALSE	TRUE	TRUE	TRUE
1	isocitric acid	1.04E-07	none	TRUE	TRUE	TRUE	FALSE	TRUE	FALSE	FALSE	TRUE	FALSE	FALSE	TRUE	TRUE	TRUE	FALSE
1	β-citryl-L-glutamic acid	4.07E-07	none	TRUE	FALSE	TRUE	FALSE	TRUE	TRUE	TRUE	TRUE	FALSE	TRUE	FALSE	TRUE	TRUE	FALSE
1	pentadecanoic acid	4.74E-06	none	TRUE	FALSE	TRUE	FALSE	TRUE	TRUE	FALSE	TRUE	FALSE	FALSE	FALSE	TRUE	TRUE	FALSE
1	phenylalanine	4.61E-05	none	TRUE	FALSE	TRUE	FALSE	TRUE	TRUE	FALSE	TRUE	FALSE	TRUE	FALSE	TRUE	TRUE	FALSE
1	2-oxoglutaric acid	5.41E-05	none	TRUE	FALSE	FALSE	FALSE	TRUE	TRUE	TRUE	TRUE	FALSE	FALSE	FALSE	FALSE	FALSE	FALSE
1	anthranilic acid	5.31E-04	nonpara	TRUE	TRUE	TRUE	TRUE	TRUE	TRUE	TRUE	TRUE	TRUE	TRUE	TRUE	TRUE	TRUE	TRUE
1	citric acid	3.04E-03	nonpara	TRUE	TRUE	TRUE	FALSE	TRUE	FALSE	FALSE	TRUE	TRUE	FALSE	TRUE	TRUE	TRUE	FALSE
2	malonic acid	3.77E-11	none	FALSE	TRUE	TRUE	TRUE	TRUE	FALSE	TRUE	TRUE	FALSE	TRUE	TRUE	FALSE	TRUE	TRUE
2	asparagine	1.28E-10	none	TRUE	TRUE	TRUE	FALSE	FALSE	TRUE	TRUE	TRUE	TRUE	FALSE	FALSE	TRUE	TRUE	TRUE
2	tyrosine	6.88E-10	none	FALSE	TRUE	TRUE	TRUE	TRUE	TRUE	TRUE	TRUE	TRUE	FALSE	FALSE	FALSE	FALSE	FALSE
2	maleic acid	8.28E-09	none	TRUE	TRUE	TRUE	TRUE	TRUE	TRUE	TRUE	TRUE	FALSE	FALSE	FALSE	TRUE	FALSE	TRUE
2	creatinine	4.20E-07	none	TRUE	FALSE	FALSE	TRUE	TRUE	TRUE	TRUE	FALSE	FALSE	FALSE	TRUE	TRUE	TRUE	TRUE
2	benzoic acid	1.07E-06	none	FALSE	FALSE	FALSE	TRUE	TRUE	FALSE	TRUE	TRUE	FALSE	FALSE	TRUE	TRUE	TRUE	TRUE
2	cinnamic acid	2.09E-06	none	FALSE	FALSE	TRUE	FALSE	FALSE	TRUE	FALSE	TRUE	TRUE	TRUE	FALSE	FALSE	TRUE	TRUE
2	octanoic acid	8.50E-06	none	FALSE	TRUE	TRUE	FALSE	TRUE	TRUE	TRUE	FALSE	TRUE	FALSE	TRUE	FALSE	TRUE	FALSE
2	coumaric acid	1.25E-05	none	FALSE	FALSE	TRUE	FALSE	TRUE	FALSE	TRUE	FALSE	TRUE	FALSE	TRUE	FALSE	TRUE	FALSE
2	2-methyloctadecanoic acid	5.66E-04	nonpara	FALSE	TRUE	TRUE	FALSE	TRUE	TRUE	TRUE	FALSE	TRUE	TRUE	TRUE	TRUE	TRUE	TRUE
2	5-oxotetrahydrofuran-2-carboxylic acid	1.38E-02	none	FALSE	FALSE	FALSE	FALSE	FALSE	FALSE	FALSE	FALSE	FALSE	FALSE	FALSE	FALSE	FALSE	FALSE
2	NADP	1.81E-02	none	FALSE	FALSE	FALSE	FALSE	FALSE	FALSE	TRUE	FALSE	FALSE	FALSE	FALSE	FALSE	FALSE	FALSE

Supplementary Table S3: Raw data for organic acid content in four replicate samples of two tissues types from three tamarillo cultivars. Values in µg/gDW.

sample	adipic	malic	syringic	vanillic	succinic	fumaric	lactic	cinnamic	benzoic	citric	malonic	aconitic	toluic	hydroxybutyric	itaconic	hydroxybenzoic
Amber peel 1	0.00095093	10.416	0.0030757	0.004	0.074	0.004688	0.097	0.00031	0.000000	48.161	0.385	4.983	0.744	0.298	50.153	0.009
Amber peel 2	0.00074418	9.703	0.0008424	0.002	0.062	0.003685	0.080	0.00012	0.000000	45.635	0.300	4.841	0.737	0.323	39.533	0.007
Amber peel 3	0.00073055	10.200	0.0013790	0.003	0.074	0.004273	0.097	0.00019	0.000000	48.383	0.340	5.605	0.775	0.434	42.084	0.007
Amber peel 4	0.00076546	10.306	0.0016336	0.003	0.070	0.004905	0.099	0.00020	0.000000	50.324	0.338	6.519	0.987	0.270	54.783	0.007
Amber pulp 1	0.00013738	13.254	0.0012622	0.006	0.215	0.009536	0.061	0.00031	0.000136	69.982	0.294	12.169	2.066	0.670	110.617	0.010
Amber pulp 2		14.175	0.0014022	0.006	0.228	0.011501	0.060	0.00030	0.000155	73.095	0.286	12.663	1.982	0.153	98.323	0.011
Amber pulp 3		14.939	0.0005492	0.005	0.239	0.010645	0.063	0.00036	0.000124	72.730	0.305	12.358	1.850	0.727	102.068	0.011
Amber pulp 4	0.00002645	12.807	0.0004162	0.005	0.208	0.011641	0.054	0.00025		68.231	0.265	12.043	1.189	0.564	91.400	0.010
Mulligan peel 1	0.00149605	4.904	0.0069832	0.004	0.125	0.003657	0.143	0.00025		41.768	0.495	4.408	1.093	0.308	39.946	0.031
Mulligan peel 2	0.00137054	4.736	0.0091193	0.005	0.124	0.003146	0.137	0.00034	0.000105	40.908	0.494	4.018	1.515	0.437	33.716	0.036
Mulligan peel 3	0.00139717	4.705	0.0086945	0.005	0.124	0.003961	0.131	0.00024		40.089	0.465	4.025	1.151	0.330	31.857	0.032
Mulligan peel 4	0.00122275	4.371	0.0075969	0.004	0.123	0.003169	0.140	0.00021		37.573	0.480	3.966	1.312	0.312	30.831	0.037
Mulligan pulp 1	0.00015758	13.911	0.0020933	0.011	0.246	0.010639	0.077	0.00053	0.000346	89.677	0.245	14.327	2.699	0.765	132.017	0.085
Mulligan pulp 2	0.00007151	10.638	0.0014045	0.009	0.190	0.010039	0.058	0.00030	0.000153	69.837	0.175	11.635	2.143	0.606	80.271	0.064
Mulligan pulp 3	0.00004265	12.400	0.0022058	0.011	0.229	0.009902	0.075	0.00044	0.000266	85.970	0.217	15.069	3.182	0.885	114.809	0.086
Mulligan pulp 4	0.00020366	12.839	0.0019672	0.012	0.255	0.011680	0.075	0.00047	0.000082	79.389	0.236	14.522	2.695	0.918	105.172	0.097
Laird large peel 1	0.00052584	7.688	0.0014637	0.003	0.083	0.009229	0.109	0.00010	0.000124	75.450	0.294	12.338	1.125	0.690	114.056	0.015
Laird large peel 2	0.00046967	5.342	0.0013052	0.002	0.063	0.006124	0.086		0.000037	56.886	0.238	9.552	0.690	0.485	75.534	0.012
Laird large peel 3	0.00089326	7.317	0.0009284	0.002	0.084	0.007750	0.110	0.00008	0.000096	76.613	0.276	12.742	1.273	0.723	88.494	0.015
Laird large peel 4	0.00036318	5.092	0.0005663	0.002	0.056	0.005489	0.075	0.00007	0.000272	60.353	0.184	9.126	0.731	0.468	71.052	0.008
Laird large pulp 1	0.00000000	8.427	0.0004170	0.010	0.157	0.023112	0.041	0.00000	0.000152	83.160	0.127	15.156	0.743	1.638	110.146	0.094
Laird large pulp 2	0.00000000	5.677		0.006	0.099	0.016643	0.027	0.00000	0.000307	58.880	0.079	9.740	1.163	1.066	63.019	0.056
Laird large pulp 3	0.00000000	5.084		0.005	0.098	0.017210	0.025	0.00000	0.000443	58.969	0.072	8.940	0.902	1.003	57.308	0.044
Laird large pulp 4	0.00000000	6.376		0.006	0.112		0.031	0.00000	0.000333	71.269	0.091	11.534	0.705	1.223	81.621	0.055
